# Assessing Skin Barrier Integrity: A Comparative Study Using Transepidermal Water Loss, Electrical Impedance Spectroscopy and Corneometry

**DOI:** 10.1111/cod.70080

**Published:** 2026-01-06

**Authors:** Charlotte Jasmin Kiani, Valentina Faihs, Claudia Kugler, Neslim Ercan, Shyami Kandage, Susanne Kaesler, Tilo Biedermann, Knut Brockow

**Affiliations:** ^1^ Department of Dermatology and Allergy Biederstein School of Medicine and Health, TUM University Hospital Rechts der Isar Munich Germany; ^2^ Odense Research Center for Anaphylaxis (ORCA), Department of Dermatology and Allergy Centre Odense University Hospital Odense Denmark

**Keywords:** allergen penetration, corneometry, electrical impedance spectroscopy, skin barrier, transepidermal water loss

## Abstract

**Background:**

Skin barrier disruption can be induced through experimental models, yet standardised comparisons are scarce. Transepidermal water loss (TEWL) and corneometry (CM) are established methods to quantify barrier impairment. Electrical impedance spectroscopy (EIS) is a novel approach, which may provide complementary information.

**Objectives:**

To compare EIS against TEWL and CM and to assess different barrier disruption models in humans in vivo.

**Methods:**

15 healthy adults (7 female, 8 male; median age 27 years) were recruited. Experiment I compared 3 adhesive tapes for tape stripping (TS). Experiment II evaluated TS, 0.5% sodium lauryl sulphate (SLS), SLS + TS, and gluten using TEWL, EIS, and CM.

**Results:**

D‐Squame tape caused stronger barrier impairment than Scotch and Tesa (TEWL *p* < 0.01, EIS *p* < 0.05). SLS + TS induced the most (8 h: TEWL *p* < 0.001; EIS *p* < 0.05; CM *p* < 0.05) and persistent disruption (24 h: TEWL *p* < 0.05, EIS *p* < 0.05), followed by TS (8 h: TEWL *p* < 0.01; EIS *p* < 0.05). SLS and aqua caused minor, non‐significant effects; gluten had no measurable impact. EIS correlated strongly with TEWL (*ρ* = −0.62, *p* < 0.0001) and CM (*ρ* = −0.61, *p* < 0.0001).

**Conclusions:**

This first human study of EIS after experimental barrier disruption demonstrated strong concordance with TEWL and CM. Combined mechanical and chemical stress best reflects real‐life barrier insults and represents a potent model.

## Graphical abstract

This study compares experimental models of human skin barrier disruption and evaluates non‐invasive assessment methods. It highlights electrical impedance spectroscopy (EIS) as a novel tool of quantifying skin barrier damage and supports methodological standardisation and an improved understanding of barrier dysfunction under real‐world conditions.

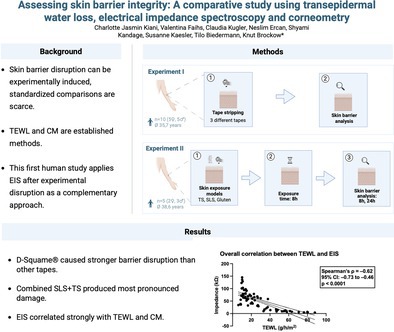

## 1 Introduction

**FIGURE 1:**
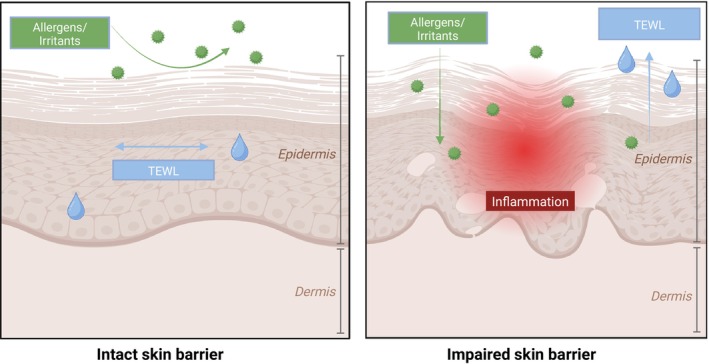
Simplified schematic skin model illustrating the importance of an intact skin barrier. (A) Physiological condition with an intact barrier, effectively protecting against transepidermal water loss and the penetration of allergens and irritants. (B) Impaired skin barrier condition leading to inflammation as well as an elevated transepidermal water loss and a higher risk for the penetration of allergens, irritants, and microbes. Created in BioRender. Köberle, M. (2025) https://BioRender.com/q41epf0.

As the largest epithelial surface of the human body, the skin is the first site of contact to physical, chemical, and microbial stressors. Even subtle perturbations in barrier integrity may lead to increased allergen penetration, elevated transepidermal water loss (TEWL), and type 2–skewed immune response (Figure 1) [1, 2]. Epithelial barrier dysfunction is increasingly recognised as a central driver of atopic diseases, including atopic dermatitis (AD), food allergy, and asthma [1, 2, 3]. In daily life, skin barrier impairment rarely results from a single insult [2]. External factors, such as mechanical abrasion, chemical detergents, and environmental exposures, often act in combination, accelerating barrier breakdown. Standardised experimental models of such barrier defects vary widely in their approach and readout, limiting our understanding of how distinct insults translate into quantifiable skin barrier dysfunction in humans [4].

Understanding barrier function requires reliable, non‐invasive assessment methods. Whilst TEWL remains the gold standard for evaluating barrier integrity, electrical impedance spectroscopy (EIS) has recently emerged as a promising alternative, offering potential advantages in terms of practicality and reduced environmental sensitivity [5, 6, 7]. Although EIS has been validated for distinguishing healthy skin from AD, its application in monitoring experimentally induced barrier disruption in humans has not been evaluated [7].

Regarding experimental models of barrier disruption, sequential tape stripping (TS) can be used to induce mechanical disruption [8]. On the other hand, chemical detergents like sodium lauryl sulphate (SLS) can alter lipid organisation and induce keratinocyte damage [9, 10]. Additionally, allergens themselves may contribute to barrier disruption at different organ sites [11, 12, 13, 14]. However, comparative data on different disruption methods and their effects on various measurement techniques remain limited.

This study aims to systematically compare different experimental models of skin barrier disruption and to evaluate the performance of different non‐invasive measurement techniques. We investigate the utility of EIS as an alternative to TEWL and corneometry (CM), compare the barrier‐disrupting potential of three adhesive tapes, and assess the effects of mechanical, chemical, and combined interventions using TEWL, EIS, and CM.

## 2 Materials and Methods

### 2.1 Study Population and Data Collection

Fifteen healthy volunteers (≥ 18 years; 7 female, 8 male; median age 27 years) with clinically healthy skin were recruited from the Department of Dermatology and Allergology, TUM Klinikum Rechts der Isar (Munich, Germany) between January and June 2025. Subjects with a history of atopic dermatitis and the use of topical preparations within 24 h before testing were excluded. All participants provided written informed consent to participate in the study. The study received approval from the local medical ethics committee (approval number 477/21 S‐NP).

### 2.2 Non‐Invasive Measurement of the Skin Barrier

All Measurements were performed under controlled environmental conditions (20°C–22°C; 40%–60% humidity) after a 15 min acclimatisation period.

#### 2.2.1 Transepidermal Water Loss (TEWL)

TEWL was measured with the AquaFlux AF200 Tewameter (Biox Systems, London, UK). Before each measurement, the probe was placed perpendicular to the skin's surface until a stable value was achieved. TEWL values are given in g/m^2^/h [6].

#### 2.2.2 Electrical Impedance Spectroscopy (EIS)

EIS was analysed using the Nevisense device (SciBase AB, Stockholm, Sweden). Prior to each measurement, 0.9% saline solution (Salvequick wound cleansing wipe; Orkla Wound Care, Solna, Sweden) was applied on the skin surface for 30 s. Measurements were performed by placing the probe vertically on the skin and retracting the outer sleeve downward for approximately 10 s [15]. As immediate output, the device provided a manufacturer‐defined impedance score (range: 1–5). In addition, the *Z*_1_ value (kΩ) was extracted post hoc for further quantitative analysis.

#### 2.2.3 Corneometry

Corneometry (CM) was assessed using the Corneometer CM825 (Courage und Khazaka electronic GmbH, Cologne, Germany). The probe was applied to the skin for 1 s. The skin humidity is indicated in arbitrary units, with one unit representing a water content of stratum corneum of 0.02 mg/cm^2^, at a measuring depth of 20 nm [16].

### 2.3 Skin Barrier Disruption

#### 2.3.1 Tape Stripping (TS)

To sequentially remove stratum corneum layers, the target area was marked (Figure S1). Adhesive tape (D‐Squame, CuDerm, Dallas, TX, USA; Scotch Magic Tape, 3 M, Saint Paul, MN, USA; Tesa Crystal Clear Tape, Tesa SE, Norderstedt, Germany) was applied with a pressure applicator (D‐Squame Pressure Instrument, CuDerm; Figure S1) for 5 s, then removed with tweezers (Figure S1). This was repeated for 20 strips at the same site, alternating removal directions to ensure uniform SC removal.

#### 2.3.2 Epicutaneous Exposure Model

For epicutaneous application, 12 mm Finn Chambers (SmartPractice, Phoenix, AZ, USA) were used. Filter discs were loaded with either 50 μL of sodium lauryl sulphate (SLS, 0.5% aqueous solution; SDS pellets > 99% purity, Carl Roth GmbH, Karlsruhe, Germany), 50 μL of purified water (Ampuwa, Fresenius Kabi, Bad Homburg, Germany; negative control), a small amount of gluten–petrolatum mixture (15.6% native gluten flour in petrolatum; vital wheat gluten, Kröner‐Stärke GmbH, Ibbenbüren, Germany; petrolatum, Vaseline, Bombastus‐Werke AG, Freital, Germany), or petrolatum alone (negative control). Chambers were occlusively fixed to the volar forearm for 8 h and residual substances gently removed with moistened swabs.

### 2.4 Experimental Setup

#### 2.4.1 Experiment I: Comparison of Adhesive Tapes

**FIGURE 2:**
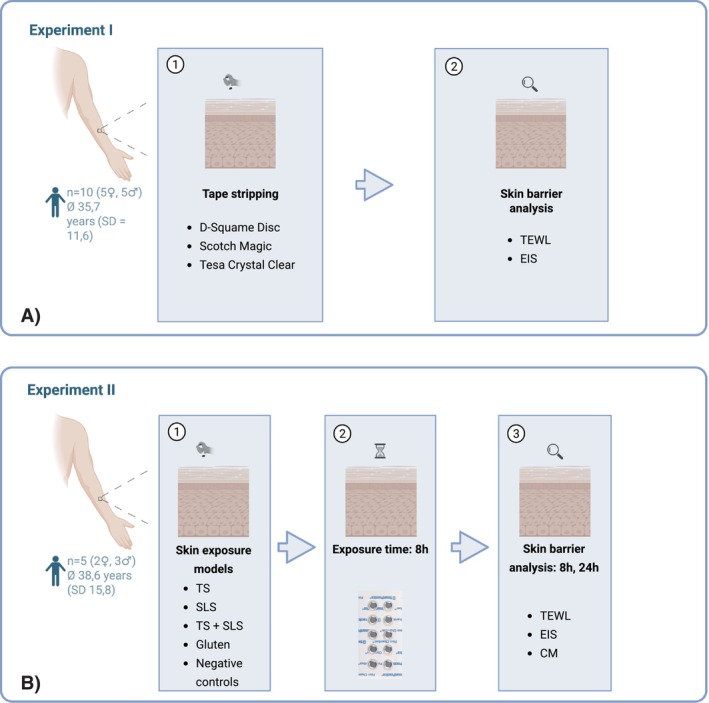
Overview of experimental models and barrier assessment protocol of the study. Created in BioRender. Köberle, M. (2025) https://BioRender.com/v2qhup9.

TS was performed on the volar forearm of 10 healthy volunteers. Three different commercially available adhesive tapes were used for barrier disruption at different sites on the forearm. Stripping was conducted in four consecutive cycles (5 strips per cycle; 20 in total), and barrier function was assessed after each stripping interval by measuring TEWL and EIS (Figure 2A).

#### 2.4.2 Experiment II: Different Types of Skin Barrier Disruption

Five healthy volunteers underwent four different skin barrier interventions with an epicutaneous exposure model on the volar forearm: (1) TS (20 tapes, D‐Squame); (2) application of SLS; (3) application of SLS followed by TS; (4) application of gluten in petrolatum. Additionally, two negative controls were applied (aqua and petrolatum). Each test substance was applied to a 1 × 1 cm area under occlusion using Finn chambers (12 mm diameter) for 8 h. Barrier function was assessed 8 h and 24 h post‐application by TEWL, EIS, and CM (Figure 2B).

### 2.5 Statistical Analysis

Statistical analysis was performed using SPSS 29.0 (IBM, Chicago, IL, USA), Microsoft Excel 16.83 (Microsoft, Redmond, WA, USA), and GraphPad Prism 10 (GraphPad Software, La Jolla, CA, USA). Patient characteristics were summarised as median ± standard deviation (SD). Data normality was assessed using the Shapiro–Wilk test. For non‐normally distributed data, Friedman's test was applied for repeated measures, followed by Dunn's post hoc test for pairwise comparisons. Analyses included within‐group changes over time and between‐group comparisons at single time points. Spearman's rank correlation assessed associations between variables. Statistical significance was set at *p* < 0.05.

## 3 Results

### 3.1 Experiment I: Comparison of Adhesive Tapes

**FIGURE 3:**
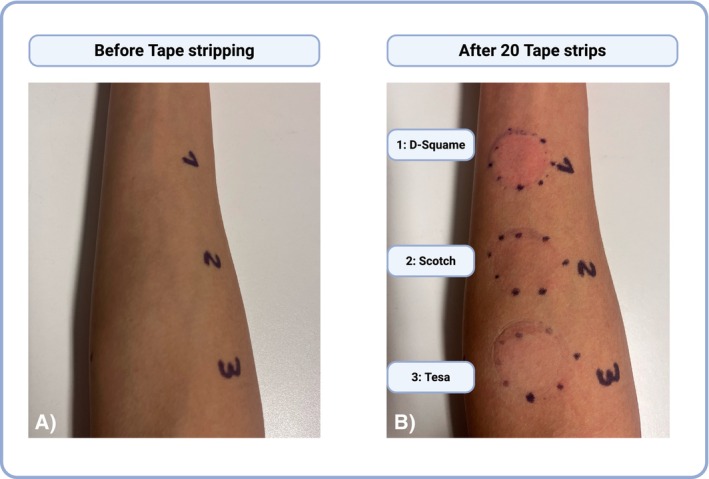
Representative images of the skin surface before (A) and after (B) tape stripping. The procedure results in a controlled disruption of the stratum corneum, used to model impaired barrier function. The degree of skin barrier disruption can be visually assessed based on erythema and the increased translucency of the epidermis. Original photograph, taken by the authors. Created in BioRender. Köberle, M. (2025) https://BioRender.com/k8sm2aj.

**FIGURE 4:**
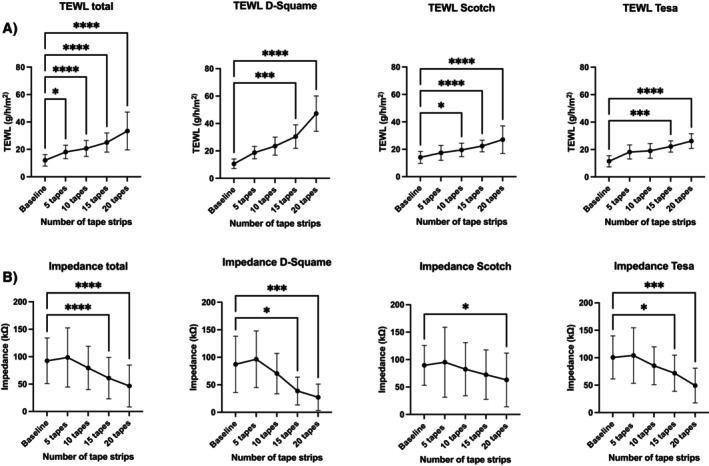
Line graph depicting (A) transepidermal water loss (g/h/m^2^) and (B) electrical impedance spectroscopy (kΩ) at baseline up to 20 tape stripped layers (intervals of 5 layers). Data are presented as absolute values (mean ± SD; *n* = 10); *p < 0.05; **p < 0.01; ***p < 0.001; ****p < 0.0001, compared to baseline.

**FIGURE 5:**
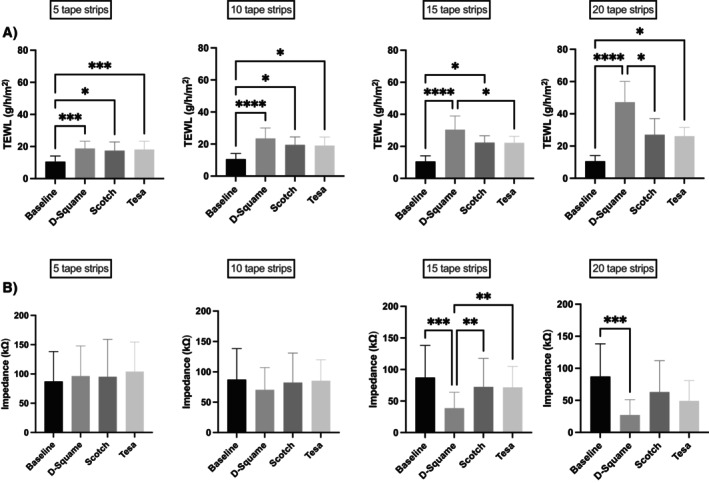
Bar charts depicting the tape material dependent differences in (A) transepidermal water loss (TEWL) and (B) electrical impedance spectroscopy (EIS). Mean TEWL (g/m^2^/h) and EIS (Ω) across different tape types (D‐Squame Disc, Scotch Magic Tape, Tesa Crystal Clear) are shown at baseline, 5, 10, 15, and 20 layers. Data are presented as absolute values (mean ± SEM, *n* = 10), with significance levels compared between the tape materials; *p < 0.05; **p < 0.01; ***p < 0.001; ****p < 0.0001.

**FIGURE 6:**
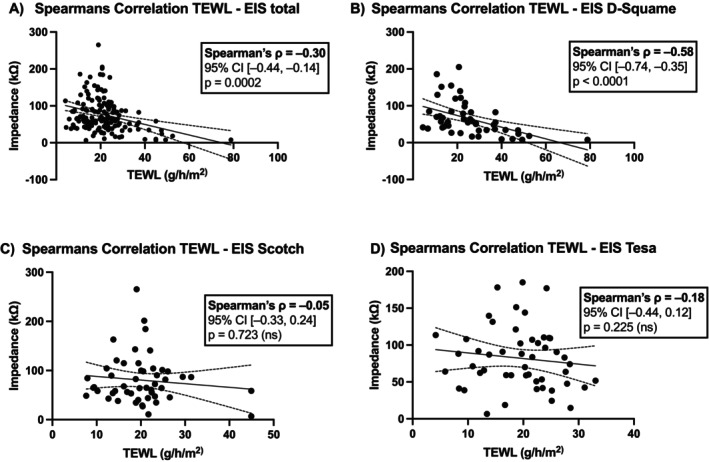
Spearman's correlation between transepidermal water loss (TEWL) in g/m^2^/h and skin impedance in kΩ across different tape types (A) all tapes (B) D‐Squame Disc, (C) Scotch Magic Tape, and (D) Tesa Crystal Clear, 5 layers, 10 layers, 15 layers and 20 layers., along with the corresponding significance values; *p < 0.05; **p < 0.01; ***p < 0.001; ****p < 0.0001.

All tapes were well tolerated, with only mild, transient reactions on the skin. Progressive stripping caused distinct, tape‐dependent barrier impairment, visually characterised by erythema and enhanced epidermal translucency, most pronounced in D‐Squame (Figure 3). Tape‐dependent barrier impairment was further confirmed by TEWL increasing from 12.0 to 33.5 g/m^2^/h across all tape types (*p* < 0.0001; baseline vs. after tape 20) and EIS decreasing from 92.5 to 46.6 kΩ across all tape types (*p* < 0.0001; baseline vs. after tape 20) (Figure 4; Table 1). D‐Squame tape produced the strongest skin barrier changes in both TEWL and EIS measurements, with significant differences compared to Tesa (TEWL: *p* < 0.01; EIS: *p* < 0.05; after tape 20) and Scotch (TEWL: *p* < 0.01; EIS: *p* < 0.05; after tape 20) (Figure 5). Notably, in EIS measurement, all tapes showed a transient, non‐significant impedance rise after the first five strips before declining (Figure 4; Figure 5). Correlation analysis further revealed a strong inverse association between TEWL and EIS for D‐Squame (*p* < 0.001; *ρ* = −0.58), a weak, non‐significant trend for Tesa (n.s.; *ρ* = −0.18), and no correlation for Scotch (n.s.; *ρ* = −0.05) (Figure 6).

**TABLE 1:** Transepidermal water loss (g/h/m^2^) and electrical impedance spectroscopy (kΩ) at baseline up to 20 tape stripped layers (intervals of 5 layers).

	Total	D‐Squame	Scotch	Tesa
Transepidermal water loss (g/h/m^2^)				
Baseline	12.02 ± 4.16	10.59 ± 3.51	14.07 ± 4.40	11.41 ± 4.09
5 layers	18.12 ± 4.89*	18.76 ± 4.52	17.41 ± 5.41	18.19 ± 5.11
10 layers	20.67 ± 5.82	23.47 ± 6.55	19.52 ± 4.91*	19.02 ± 5.37
15 layers	25.02 ± 6.96	30.44 ± 8.52***	22.39 ± 4.26	22.24 ± 4.07***
20 layers	33.46 ± 13.79	47.21 ± 12.91	27.02 ± 10.04	26.15 ± 5.40
Electrical impedance spectroscopy (kΩ)				
Baseline	92.45 ± 41.61	87.23 ± 51.10	89.52 ± 36.31	100.6 ± 39.19
5 layers	98.54 ± 53.83	96.39 ± 51.51	95.21 ± 63.84	104.0 ± 50.58
10 layers	79.39 ± 39.50	70.36 ± 36.72	82.40 ± 48.49	85.39 ± 34.40
15 layers	60.97 ± 37.73	38.67 ± 25.22*	72.46 ± 45.07	71.78 ± 33.04*
20 layers	46.55 ± 38.14	27.31 ± 23.89***	63.03 ± 48.93*	49.31 ± 31.62***

*Note:* Data are presented as absolute values (mean ± SD; *n* = 10).

* *p* < 0.05.

*** *p* < 0.001.

### 3.2 Experiment II: Different Types of Skin Barrier Disruption

**FIGURE 7:**
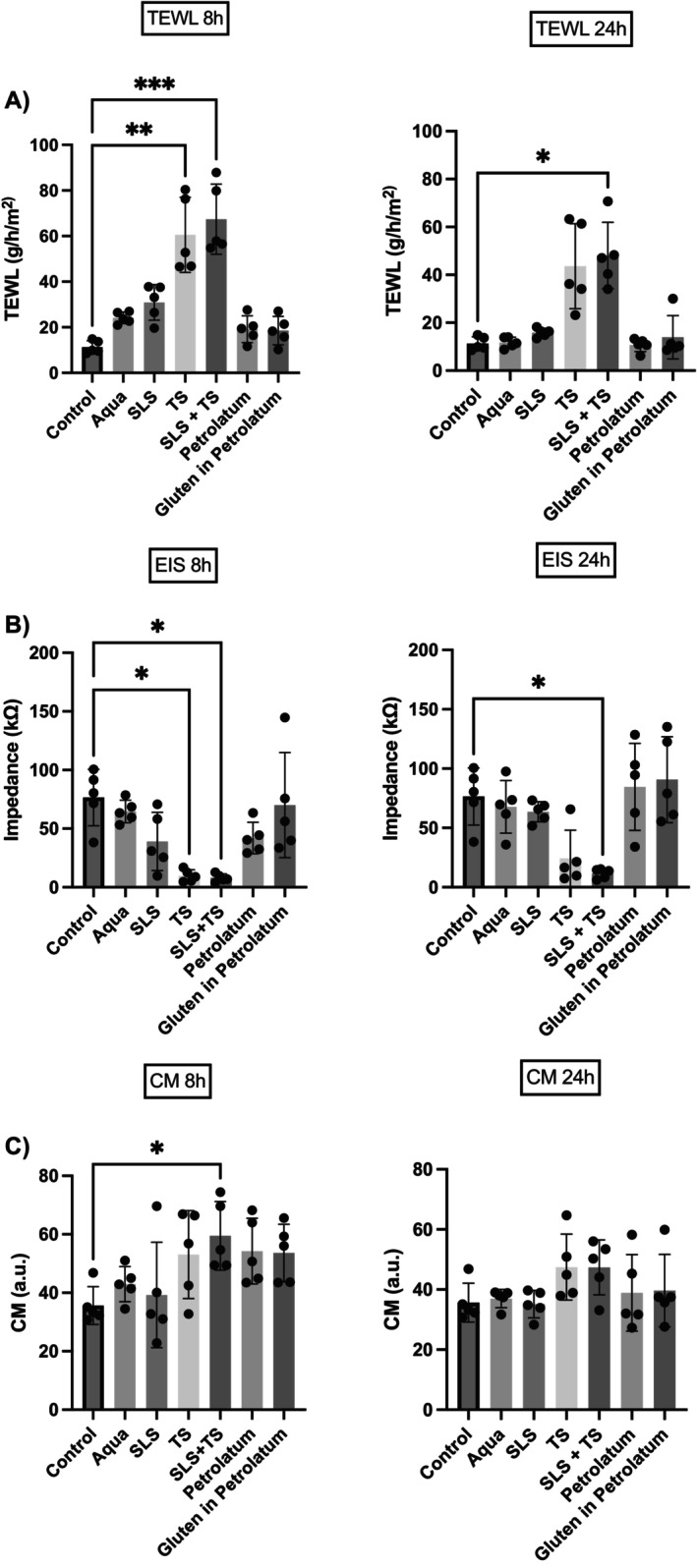
Bar charts depicting the effects of different skin barrier interventions measured with (A) transepidermal water loss (TEWL, B) electrical impedance spectroscopy (EIS) and (C) corneometry (CM) after 8 and 24 h. Data are presented as absolute values (mean ± SEM, *n* = 5); *p < 0.05; **p < 0.01; ***p < 0.001; ****p < 0.0001.

**FIGURE 8:**
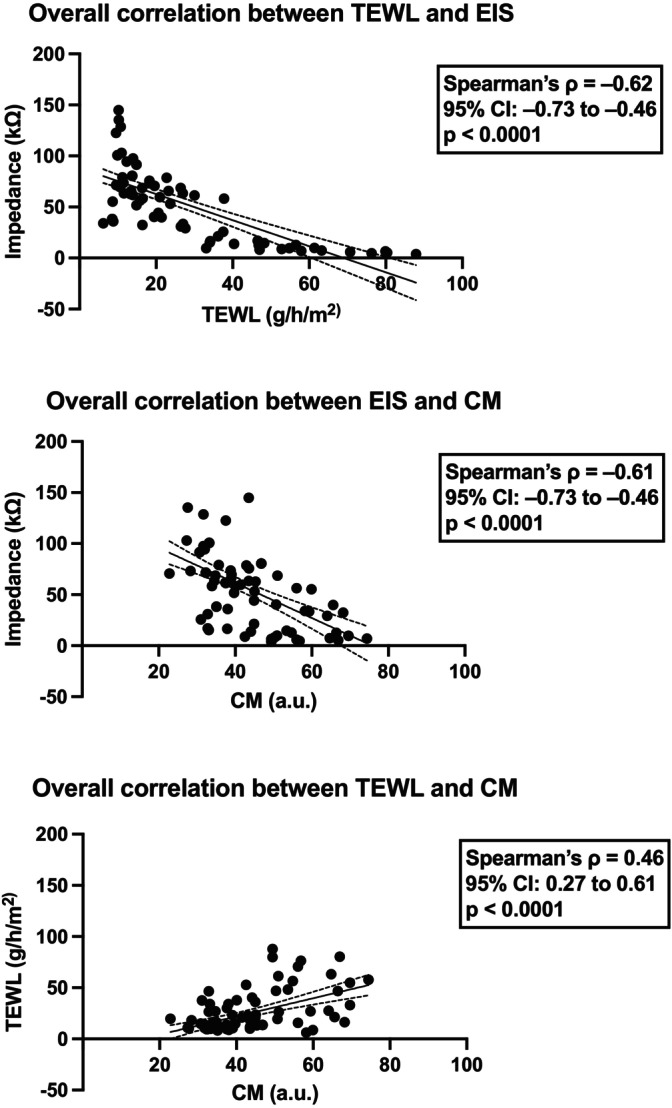
Spearman's correlation between transepidermal water loss (TEWL) electrical impedance spectroscopy (EIS) and corneometry (CM). *p < 0.05; **p < 0.01; ***p < 0.001; ****p < 0.0001.

The different methods of skin barrier intervention were well tolerated by all subjects. The three non‐invasive methods (TEWL, EIS, CM) detected significant changes following skin barrier disruption with TS alone and TS with SLS (Figure 7, Table 2). After 8 h, TEWL was highest for the combination of SLS + TS (67.4 g/m^2^/h; *p* < 0.001 vs. baseline), followed by TS alone (60.6 g/m^2^/h; *p* < 0.01 vs. baseline) and SLS (30.9 g/m^2^/h; n.s. vs. baseline). Aqua alone induced a slight, non‐significant trend towards TEWL increase (24.2 g/m^2^/h), whilst petrolatum and gluten–petrolatum remained close to control levels. At 24 h, TEWL declined in all groups but remained elevated in SLS + TS (48.1 g/m^2^/h; *p* < 0.05 vs. baseline), indicating delayed recovery (Figure 7A, Table 2). EIS showed inverse data compared to TEWL. At 8 h, EIS showed a marked decrease in SLS + TS (8.0 kΩ; *p* < 0.05 vs. baseline) and TS (9.7 kΩ; *p* < 0.05 vs. baseline). Smaller, non‐significant reduction trends were observed for SLS (39.1 kΩ) and petrolatum (42.0 kΩ), whilst aqua (64.7 kΩ) and gluten–petrolatum (70.1 kΩ) remained close to baseline. At 24 h, just SLS + TS (11.6 kΩ; *p* < 0.05 vs. baseline) showed sustained reduced values (Figure 7B, Table 2). CM revealed hydration at 8 h for SLS + TS (59.5 a.u.; *p* < 0.05 vs. baseline), but without persistent differences at 24 h (Figure 7C, Table 2). Overall, SLS + TS caused the strongest and most persistent barrier impairment, followed by TS alone, whilst SLS alone led to a non‐significant trend towards changes in skin barrier measurements and gluten did not measurably affect barrier integrity. Spearman's analysis confirmed the relationships between measurement methods (Figure 8). In concordance with Experiment I, TEWL and EIS showed a strong, significant correlation (*p* < 0.0001; *ρ* = −0.62; Figure 8A), as did EIS and CM (*p* < 0.0001; *ρ* = −0.61; Figure 8B) as well as TEWL and CM (*p* < 0.0001; *ρ* = 0.46; Figure 8C).

**TABLE 2:** Transepidermal water loss (g/h/m^2^), electrical impedance spectroscopy (kΩ) and corneometry (a.u.) 8 and 24 h after intervention compared to baseline.

	Aqua	SLS	TS	SLS + TS	Petrolatum	Gluten in petrolatum
Baseline	Transepidermal water loss (g/h/m^2^)					
11.32 ± 2.79						
8 h	24.16 ± 2.52	30.90 ± 7.80	60.56 ± 16.50**	67.41 ± 15.36***	19.14 ± 5.88	18.53 ± 6.28
24 h	11.73 ± 2.23	15.88 ± 1.79	43.63 ± 17.75	48.08 ± 13.88*	10.70 ± 2.73	13.93 ± 9.01
Baseline	Electrical impedance spectroscopy (kΩ)					
76.52 ± 24.06						
8 h	64.71 ± 9.59	39.06 ± 24.89	9.72 ± 5.12*	8.01 ± 3.40*	42.00 ± 13.43	70.06 ± 44.87
24 h	67.74 ± 22.20	63.60 ± 8.54	24.18 ± 23.39	11.57 ± 4.19*	84.60 ± 36.73	90.71 ± 36.36
Baseline	Corneometry (a.u.)					
35.62 ± 6.45						
8 h	42.96 ± 5.98	39.26 ± 18.04	53.08 ± 15.05	59.50 ± 11.74*	54.26 ± 11.24	53.60 ± 9.80
24 h	36.98 ± 3.02	35.12 ± 4.57	47.46 ± 10.96	47.38 ± 9.13	38.90 ± 12.72	39.64 ± 12.04

*Note:* Data are presented as absolute values (mean ± SD; *n* = 5).

Abbreviations: SLS, sodium lauryl sulphate; TS, tape stripping.

* *p* < 0.05.

** *p* < 0.01.

*** *p* < 0.001, compared to baseline.

## 4 Discussion

This study systematically compared different experimental skin barrier disruption models and assessed their effects using three non‐invasive techniques: transepidermal water loss (TEWL), electrical impedance spectroscopy (EIS), and corneometry (CM). To our knowledge, this is the first study demonstrating that EIS is a valid method to study skin barrier impairment following experimental disruption in humans, showing a strong correlation with TEWL and CM under identical conditions. Additionally, this study highlights differences between adhesive tapes, the superiority of TS over SLS, and the additional effect of combined mechanical and chemical stress for barrier disruption.

In this study, EIS correlated strongly and inversely with TEWL and CM (both *p* < 0.0001) across all experiments. Still, TEWL is the current gold standard in assessing skin barrier changes, whereas CM is widely used as a complementary method to evaluate hydration status [6, 17]. To our knowledge, the application of EIS in assessing barrier disruption in humans has not been reported to date [18]. Previous studies have shown that EIS is able to differentiate between healthy skin and AD and can detect skin barrier disruption in murine models [7, 18, 19, 20]. EIS potentially provides additional information on the structural integrity of the stratum corneum and exhibits reduced susceptibility to environmental influences compared to TEWL measurements [5, 6, 18, 21]. Notably, EIS showed a trend towards an initial increase after five TS before subsequently decreasing, suggesting that EIS may be sensitive to different structural alterations than TEWL. EIS is dependent on cell cohesion and lipid composition, whereas TEWL reflects water flux across the epidermis. Taken together, these findings highlight the potential of EIS as a sensitive and complementary method for barrier assessment that may offer practical advantages over conventional TEWL and underscores its potential as an early biomarker for barrier damage.

Deterioration of barrier function by TS was strongly dependent on the adhesive tape material. D‐Squame tape induced a statistically significant superior barrier disruption compared to Tesa and Scotch. The present study extends these findings by showing an inverse correlation between TEWL and EIS with D‐Squame, but no other tapes, indicating that tape choice is not only a key determinant in the magnitude of disruption, but also in the sensitivity of measurement modalities. Our findings are consistent with another study showing that tape material critically affects stratum corneum removal. In that study, however, different tapes were compared and significant differences only emerged after 22 strippings [22]. Thus, tape choice may not only determine the extent of barrier disruption, but also how rapidly barrier damage develops.

This study systematically compared mechanical (tape stripping), chemical (sodium lauryl sulphate), and combined (tape stripping + sodium lauryl sulphate) models of skin barrier disruption. The combination of TS + SLS resulted in the most consistent and severe barrier disruption across all parameters, followed by TS and non‐significant trends for SLS alone. Comparable additive effects on TEWL have been reported by Fluhr et al., who combined SLS exposure to mechanical irritation by hand brushes and observed greater disruption than with either stressor alone [23]. Such combined models may also more closely mimic real‐life conditions, in which barrier damage is rarely caused by a single factor but typically results from the interplay of multiple stressors, such as detergents, friction, and occlusion. SLS alone induced only minimal, non‐significant changes in skin barrier parameters. It is a well‐established anionic surfactant, known to disrupt the skin barrier by altering stratum corneum lipid organisation and exerting direct cytotoxicity on keratinocytes [9, 10, 23]. Its barrier‐compromising effects, including upregulation of inflammatory mediators and dysregulation of lipid metabolism, have been shown to increase over prolonged exposures, typically becoming more pronounced after 48–96 h [23, 24]. The absence of stronger effects in our short‐term model is therefore in line with previous evidence.

In this study, short‐term occlusion with aqua led to a modest, though not statistically significant change in skin barrier parameters. Whilst previous work demonstrated that prolonged occlusion or occlusion after prior barrier damage can influence recovery dynamics, our results may suggest that *brief* water occlusion on healthy, intact skin could already lead to biophysical changes [23, 25, 26]. Transient barrier impairment may occur through hydration‐induced swelling and loosening of corneocyte cohesion [23]. Thus, even inert‐appearing control conditions could subtly modify skin barrier measurements and should be carefully considered in experimental design.

Previous studies have demonstrated that allergens themselves can contribute to barrier disruption [11, 12, 13, 27]. In AD patients, it was shown that house dust mite proteins are able to directly impair the skin barrier through an intrinsic protease activity, downregulation of genes involved in cell–cell adhesion, the induction of hyperproliferation, and also through the disturbance of keratinocyte differentiation [11]. For gliadin, a wheat‐derived gluten protein playing a central role in wheat allergy, it has been shown that fragments of this protein are able to modulate intestinal epithelial permeability [12, 14, 28]. Despite its described impact on intestinal permeability, in this study, topical application of gluten in petrolatum for 8 h did not induce relevant changes in skin barrier parameters, suggesting no relevant barrier‐disruptive potential under the tested conditions. These findings contribute to the limited evidence regarding the direct effects of food‐derived proteins on the skin barrier and should be further assessed in future studies.

The study is limited by its relatively small sample size, which may reduce the generalizability of the findings. Furthermore, interindividual variability was evident across all methods. This suggests that individual skin barrier characteristics may differ substantially even amongst healthy individuals. One possible contributing factor could be the presence of subclinical susceptibilities to barrier disruption, such as filaggrin mutations or changes in the skin microbiome [29, 30]. Such underlying variability may influence both baseline barrier function and the skin's response to external stimuli. Moreover, the present study focused exclusively on short‐term barrier effects, with measurements conducted either immediately after intervention (Experiment I) or up to 24 h post‐application (Experiment II). As a result, potential long‐term consequences such as sustained inflammation or delayed barrier regeneration could not be assessed.

In this in vivo human systematic comparison of EIS with established methods for monitoring experimentally induced skin barrier disruption in humans, EIS demonstrated strong inverse correlations with TEWL and CM. EIS captured early and subtle structural changes not detected by TEWL measurement, suggesting it as a valuable tool to quantify barrier impairment in humans and as a viable complement to TEWL. The study demonstrated that mechanical, chemical, and occlusive stressors are able to induce distinct and measurable effects on the human skin barrier. D‐Squame tape showed superior barrier disruption efficacy compared to other commercially available tapes. The combination of SLS and TS produced the most consistent and severe disruption across all biophysical parameters. This approach may reflect real‐life conditions most accurately, where mechanical stress frequently coincides with occlusion and chemical exposure such as detergents [2, 23]. Taken together, this study provides novel evidence supporting EIS as a valuable and sensitive tool for assessing skin barrier impairment. Additionally, the findings contribute to the methodological standardisation of experimental human models for skin barrier research. By linking experimental findings to lifelike exposure scenarios, this work enhances the conceptual understanding of how different stressors may interact to compromise skin integrity.

## Author Contributions

**Charlotte Jasmin Kiani:** investigation, methodology, visualization, writing – original draft, writing – review and editing, software, formal analysis, data curation, conceptualization. **Valentina Faihs:** conceptualization, formal analysis, methodology, writing – review and editing, writing – original draft, validation. **Claudia Kugler:** conzeptualization, methodology, validation, and writing – review and editing. **Neslim Ercan:** formal analysis, methodology and writing – review and editing. **Shyami Kandae:** formal analysis, methodology and writing – review and editing. **Susanne Kaesler:** supervision, methodology and writing – review and editing. **Tilo Biedermann:** supervision, funding aquisition, resources, writing – review and editing. **Knut Brockow:** project administration, funding aquisition, resources, supervision, conzeptualization, methodology, validation, writing – original draft as well as writing – review and editing.

## Funding

This work was supported by the German Federal Ministry of Research, Technology and Space (BMTR) as part of the ABROGATE project (grant number 01EA2106A) awarded to KB, by the Clinician Scientist Program TRIAL of the German Society for Allergy and Clinical Immunology (Deutsche Gesellschaft für Allergologie und klinische Immunologie, DGAKI) to VF as well as by the German Research Foundation (Deutsche Forschungsgemeinschaft, DFG) within the RTG 2668 framework (Project A2, Project A3; Project‐ID: 435874434).

## Ethics Statement

The study was approved by the Ethics Committee of the Technical University of Munich (approval number 477/21 S‐NP). Informed consent was obtained from all patients involved in the study. In addition, written consent for publication was obtained for any photographs.

## Conflicts of Interest

The authors declare no conflicts of interest.

## Supporting information


**Figure S1:** Tape stripping procedure. (A) Marking of the target skin area. (B) Application of the adhesive tape to the skin surface. (C) Standardised pressure application using a controlled‐pressure device. (D) Careful removal of the tape using tweezers. Original photograph, taken by the authors.
**Table S1:** Transepidermal water loss (g/m^2^/h) and electrical impedance spectroscopy (kΩ) at baseline up to 20 tape stripped layers (intervals of 5 layers). Data are presented as absolute values (mean ± SD; *n* = 10); *p < 0.05; **p < 0.01; ***p < 0.001; ****p < 0.0001, compared to baseline.
**Table S2:** Transepidermal water loss (g/m^2^/h), electrical impedance spectroscopy (kΩ) and corneometry (a.u.) 8 and 24 h after intervention compared to baseline. Data are presented as absolute values (mean ± SD; *n* = 5); *p < 0.05; **p < 0.01; ***p < 0.001; ****p < 0.0001, compared to baseline. SLS = Sodium lauryl sulphate, TS = Tape stripping.

## Data Availability

The data that supports the findings of this study are available in the Supporting Information of this article.
