# Supplementary material for: Assessing Skin Barrier Integrity: A Comparative Study Using Transepidermal Water Loss, Electrical Impedance Spectroscopy and Corneometry
Source: Contact Dermatitis. 2026 Jan 6;94(4):327–37. doi: 10.1111/cod.70080 (PMC12956423; doi:10.1111/cod.70080)
Supplement: Supplementary file 1 — Figure S1: Tape stripping procedure. (A) Marking of the target skin area. (B) Application of the adhesive tape to the skin surface. (C) Standardised pressure application using a controlled‐pressure device. (D) Careful removal of the tape using tweezers. Original photograph, taken by the authors. Table S1: Transepidermal water loss (g/m2/h) and electrical impedance spectroscopy (kΩ) at baseline up to 20 tape stripped layers (intervals of 5 layers). Data are presented as absolute values (mean ± SD; n = 10); *p < 0.05; **p < 0.01; ***p < 0.001; ****p < 0.0001, compared to baseline. Table S2: Transepidermal water loss (g/m2/h), electrical impedance spectroscopy (kΩ) and corneometry (a.u.) 8 and 24 h after intervention compared to baseline. Data are presented as absolute values (mean ± SD; n = 5); *p < 0.05; **p < 0.01; ***p < 0.001; ****p < 0.0001, compared to baseline. SLS = Sodium lauryl sulphate, TS = Tape stripping. [file COD-94-327-s001.docx]

**Supplementary Material**

| **Transepidermal water loss (g/h/m^2^)** | | | | |
| --- | --- | --- | --- | --- |
| Baseline  5 layers  10 layers  15 layers  20 layers | **Total**  12.02 ± 4.16  18.12 ± 4.89*  20.67 ± 5.82****  25.02 ± 6.96****  33.46 ± 13.79**** | **D-Squame**  10.59 ± 3.51  18.76 ± 4.52  23.47 ± 6.55  30.44 ± 8.52***  47.21 ± 12.91**** | **Scotch**  14.07 ± 4.40  17.41 ± 5.41  19.52 ± 4.91*  22.39 ± 4.26****  27.02 ± 10.04**** | **Tesa**  11.41 ± 4.09  18.19 ± 5.11  19.02 ± 5.37  22.24 ± 4.07***  26.15 ± 5.40**** |
| **Electrical impedance spectroscopy (kΩ)** | | | | |
| Baseline  5 layers  10 layers  15 layers  20 layers | **Total**  92.45 ± 41.61  98.54 ± 53.83  79.39 ± 39.50  60.97 ± 37.73****  46.55 ± 38.14**** | **D-Squame**  87.23 ± 51.10  96.39 ± 51.51  70.36 ± 36.72  38.67 ± 25.22*  27.31 ± 23.89*** | **Scotch**  89. 52 ± 36.31  95.21 ± 63.84  82.40 ± 48.49  72.46 ± 45.07  63.03 ± 48.93* | **Tesa**  100.6 ± 39.19  104.0 ± 50.58  85.39 ± 34.40  71.78 ± 33.04*  49.31 ± 31.62*** |

**Supplementary Table 1.** Transepidermal water loss (g/m^2^/h) and electrical impedance spectroscopy (kΩ) at baseline up to 20 tape stripped layers (intervals of 5 layers). Data are presented as absolute values (mean ± SD; n= 10); *p<0.05; **p<0.01; ***p<0.001; ****p<0.0001, compared to baseline.

| **Baseline**  11.32 ± 2.79 | **Transepidermal water loss (g/h/m^2^)** | | | | | |
| --- | --- | --- | --- | --- | --- | --- |
| 8h  24h | **Aqua**  24.16 ± 2.52  11.73 ± 2.23 | **SLS**  30.90 ± 7.80  15.88 ± 1.79 | **TS**  60.56 ± 16.50**  43.63 ± 17.75 | **SLS + TS**  67.41 ± 15.36***  48.08 ± 13.88* | **Petrolatum**  19.14 ± 5.88  10.70 ± 2.73 | **Gluten**  18.53 ± 6.28  13.93 ± 9.01 |
| **Baseline**  76.52 ±  24.06 | **Electrical impedance spectroscopy (kΩ)** | | | | | |
| 8h  24h | **Aqua**  64.71 ± 9.59  67.74 ± 22.20 | **SLS**  39.06 ± 24.89  63.60 ± 8.54 | **TS**  9.72 ± 5.12*  24.18 ± 23.39 | **SLS + TS**  8.01 ± 3.40*  11.57 ± 4.19* | **Petrolatum**  42.00 ± 13.43  84.60 ± 36.73 | **Gluten**  70.06 ± 44.87  90.71 ± 36.36 |
| **Baseline**  35.62 ± 6.45 | **Corneometry (a.u.)** | | | | | |
| 8h  24h | **Aqua**  42.96 ± 5.98  36.98 ± 3.02 | **SLS**  39.26 ± 18.04  35.12 ± 4.57 | **TS**  53.08 ± 15.05  47.46 ± 10.96 | **SLS + TS**  59.50 ± 11.74*  47.38 ± 9.13 | **Petrolatum**  54.26 ± 11.24  38.90 ± 12.72 | **Gluten**  53.60 ± 9.80  39.64 ± 12.04 |

**Supplementary Table 2.** Transepidermal water loss (g/m^2^/h), electrical impedance spectroscopy (kΩ) and corneometry (a.u.) 8 and 24 hours after intervention compared to baseline. Data are presented as absolute values (mean ± SD; n= 5); *p<0.05; **p<0.01; ***p<0.001; ****p<0.0001, compared to baseline. SLS = Sodium lauryl sulfate, TS = Tape stripping.

**Supplementary Figure 1.** **Tape stripping procedure.**

A) Marking of the target skin area. B) Application of the adhesive tape to the skin surface. C) Standardized pressure application using a controlled-pressure device. D) Careful removal of the tape using tweezers. Original photograph, taken by the authors.
